# Hijacking host RNA polymerase for processive in situ mutagenesis

**DOI:** 10.1016/j.synbio.2026.09.002

**Published:** 2026-09-18

**Authors:** Yuou Sheng, Huizhen Ni, Shihao Yang, Wenliang Hao, Chong Zhang

**Affiliations:** a MOE Key Laboratory for Industrial Biocatalysis, Institute of Biochemical Engineering, Department of Chemical Engineering, Tsinghua University, Beijing, 100084, China; b School of Life Sciences, Tsinghua University, Beijing, 100084, China; c College of Food Science and Technology, Nanjing Agricultural University, Nanjing, 210095, China; d Beijing Key Laboratory of Recombinant Proteins and Biomanufacturing, Tsinghua University, Beijing, 100084, China; e State Key Laboratory of Green Biomanufacturing, Tsinghua University, Beijing, 100084, China; f Center for Synthetic and Systems Biology, Tsinghua University, Beijing, 100084, China

**Keywords:** Directed evolution, Targeted mutagenesis, Transcription-coupled mutagenesis, *In situ* evolution, Microbial chassis

## Abstract

Targeted mutagenesis is a crucial tool for in vivo continuous evolution. However, existing methods for long-range targeted mutagenesis in vivo often face limitations such as low efficiency, poor host compatibility, operational complexity, and uneven distribution of mutations across these windows. In this study, we developed a novel in vivo Targeted Assisted Mutagenesis via Endogenous RNAP tool (TAMER) by functionally fusing the endogenous ω subunit of RNAP with a deaminase and dCas9. This tool achieved a mutagenesis efficiency of 1.28 × 10^−3^ substitutions per base (s.p.b.), which represents a 4.2 × 10^5^-fold increase over the natural mutation rate. Additionally, this method enables uniform mutation distribution, an even mutation rate, a broad mutagenesis window, low off-target effects, and cross-host portability. Overall, TAMER offers a simple, efficient, and broadly applicable strategy for in vivo targeted long-range mutagenesis.

## Graphical abstract

**Figure:**
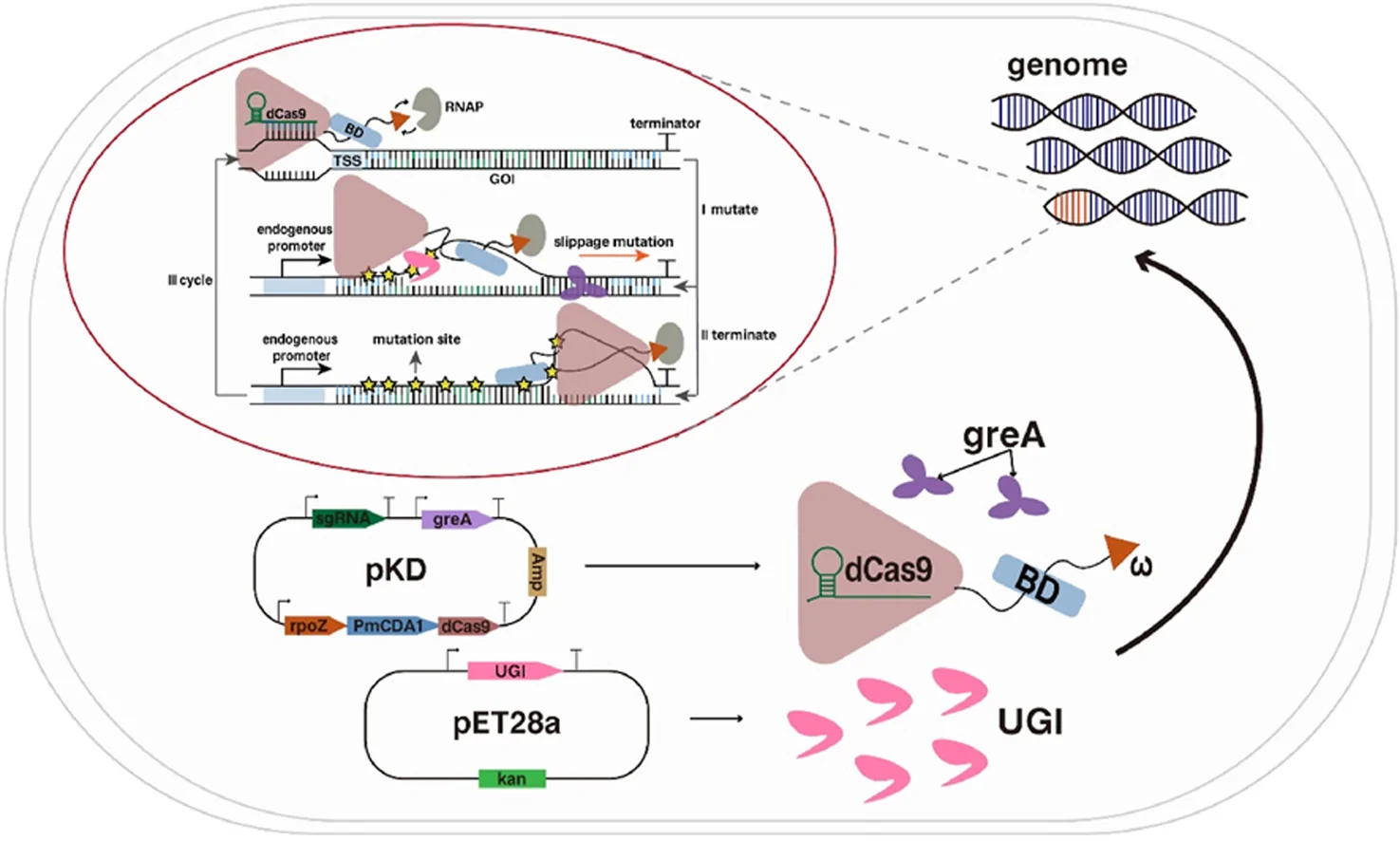

## 1 Introduction

Directed evolution represents a powerful and extensively employed strategy for the engineering of proteins, metabolic pathways, and entire genomes, aimed at achieving functional optimization or novel functionalities [1,2]. Among the available approaches, in vivo targeted mutagenesis has become a prominent experimental method due to its capability to efficiently and precisely elevate mutation rates within specific genomic loci. This acceleration facilitates the compression of evolutionary timelines and expedites the isolation of beneficial mutations. As such, the approach holds significant value for the rapid and high-throughput exploration of sequence mutational diversity, the systematic mapping of sequence–function relationships, and the efficient acquisition of targeted biological phenotypes.

Currently, a variety of long-range mutagenesis tools coupled with replication or transcription processes have been successfully developed. The most classic example is the OrthoRep [3] system based on orthogonal DNA replication in yeast, which can specifically perform error-prone replication on linear plasmids without affecting the mutation rate of the genome. Subsequently, OrthoRep systems for bacteria were successively developed, expanding the applicability of this type of system. Nevertheless, this type of system still faces challenges such as operational complexity and host portability issues. Another classical bacterial system for genome-targeted mutagenesis based on error-prone DNA replication is EvolvR [4], but it is limited by a narrow mutational window and high off-target effects. While these issues were later resolved by the development of OMEGA-R [5] and CTRLE [6] systems, the latter still suffer from low and uneven mutation rates. A new targeted mutagenesis tool based on the Cas3 helicase process (CoMuTER [7]) also suffers from the problem of uneven mutation frequency. Tools based on orthogonal transcription (e.g., MutaT7 [8], OTM [9]) employ deaminases fused to heterologous phage RNAPs to introduce C > T or A > G mutations within single-stranded DNA regions of transcription-generated R-loops. Such systems have been successfully applied in various microorganisms, including multiple model bacteria and yeasts, for protein functional evolution and metabolic pathway optimization. Nonetheless, these approaches generally require the pre-insertion of specific promoters upstream of the target gene, complicating experimental procedures and limiting their utility in genetically recalcitrant hosts. In summary, there remains a need for a novel in vivo targeted mutagenesis tool characterized by high and uniform mutation rate, a broad mutagenic window, robust host compatibility, and operational simplicity.

In bacteria, transcription is initiated following the assembly of RNAP upon recognition of promoter regions. This process involves σ factor binding to the −35 and −10 promoter elements, leading to the recruitment and assembly of the core enzyme subunits (α, β, β′, and ω) to form a closed complex [10,11]. During transcription, RNAP moves along the template strand within a transcription bubble to synthesize mRNA [11]. As mRNA pairs with the template strand, the non-template strand is exposed as ssDNA, allowing mutations to be introduced via deaminase activity during transcription. Based on this principle, we developed an Targeted Assisted Mutagenesis via Endogenous RNAP tool, termed TAMER. Guided by sgRNA, a fusion protein of ω subunit, deaminase, and dCas9 is recruited upstream of the target gene to reassemble a functional RNAP and initiate transcription. During transcription, the deaminase acts on the ssDNA within the R-loop structure to introduce targeted mutations. To enhance mutagenesis efficiency, factors influencing transcription initiation and elongation were systematically investigated. Transcription initiation was modulated by adjusting sgRNA targeting positions and introducing sgRNA mismatches to facilitate transcriptional slippage. Furthermore, transcription elongation factors and uracil glycosylase inhibitors were introduced to sustain transcription progression and synchronize it with deamination activity. Performance evaluations revealed that TAMER achieved a targeted mutagenesis frequency of 1.28 × 10^−3^ s.p.b. in *Escherichia coli*. Moreover, it enabled uniform mutagenesis across the entire length of target genes in both *E*. *coli* and *C*. *glutamicum*, with G→A and C→T transitions as the predominant mutation types. Collectively, TAMER exhibits high mutagenesis efficiency, a broad mutagenic window, and cross-host portability.

## 2 Results

**Fig. 1:**
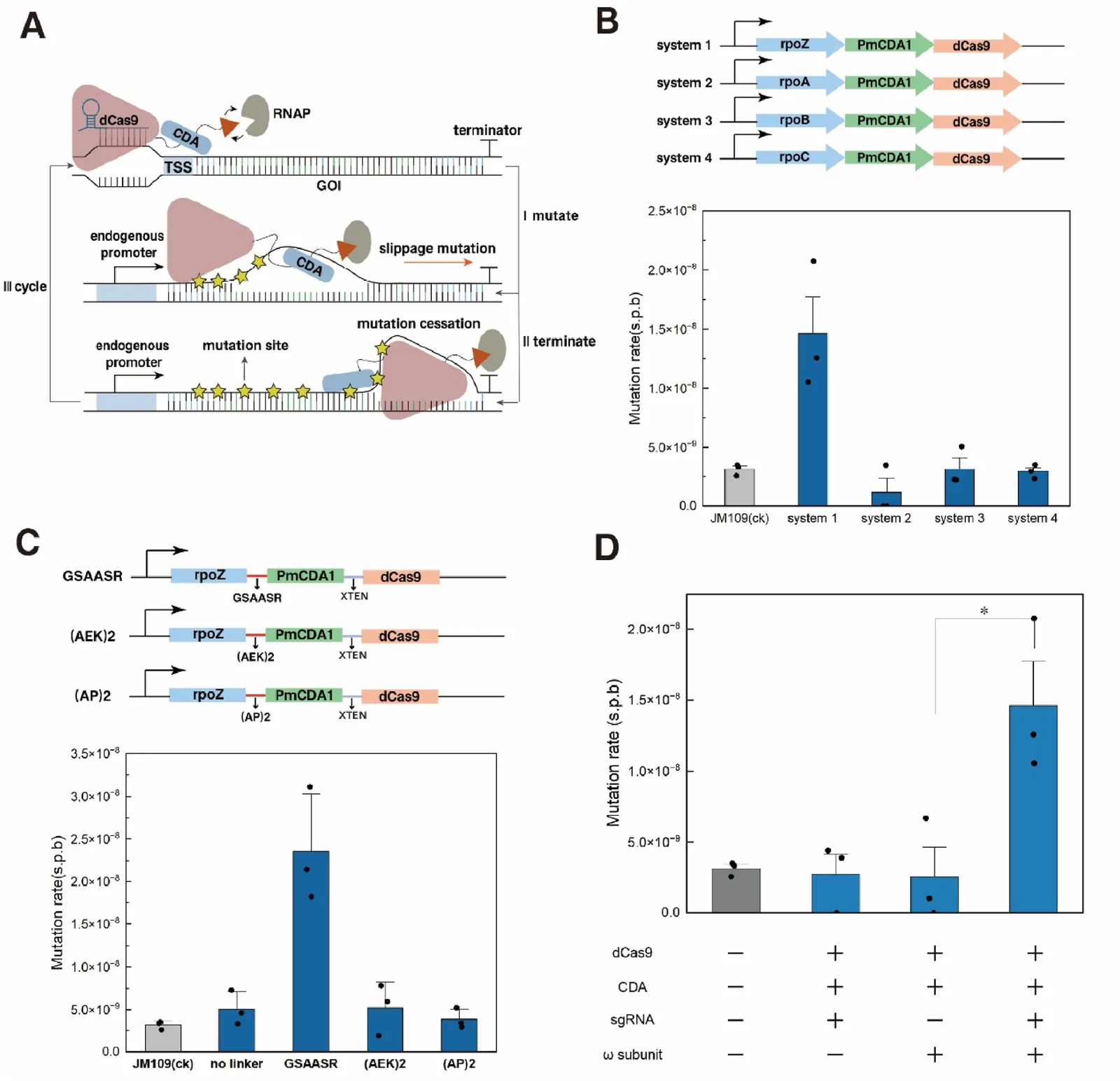
**Design of the TAMER.** (A) Schematic design and mutagenesis mechanism of the TAMER. (B) Validation of mutagenesis frequencies of different TAMER constructs. (C) Influence of linker length on mutagenesis efficiency of TAMER. (D) Mutation rates of TAMER under indicated conditions. “+” denotes inclusion of the specified component; “−” indicates its absence. Data are presented as mean (bar), standard error (error bar), and individual data points (black dots). n = 3 independent biological replicates. NS, not significant (P > 0.05). *P < 0.05, **P < 0.01, ***P < 0.001 and ****P < 0.0001.

First, we constructed a mutagenesis tool by fusing RNAP subunits with dCas9 and a deaminase, potentially enabling the recruitment of a functional RNAP complex upstream of the target gene, where the deaminase would catalyze C-to-T mutations on the non-template strand during transcription (Fig. 1A). To assess the feasibility of achieving targeted mutagenesis based on transcription slippage in *E*. *coli*, four distinct transcription-coupled mutagenesis systems were engineered (Fig. 1B). Using sgRNA to target the four systems to the *rpsE* gene (mutations in *rpsE* can confer spectinomycin resistance to *E. coli*), the mutagenesis efficiencies were calculated and compared through fluctuation analysis. Notably, the fusion of the deaminase with the ω subunit of RNAP yielded the most pronounced mutagenic effect, achieving a mutation rate approximately 6.7-fold higher than the spontaneous mutation rate (Fig. 1B). Evaluation of three linkers of varying lengths (GSAASR, (AEK)_2_, and (AP)_2_) revealed a positive correlation between linker length and mutagenesis efficiency (Fig. 1C). To verify that the observed mutations were indeed driven by the synergistic action of transcription slippage and deaminase activity, control experiments were performed using systems lacking either the ω subunit or the sgRNA. The results robustly confirmed the validity of the proposed mutagenic mechanism (Fig. 1D). Additionally, growth curve analysis indicated that TAMER exerted no significant inhibitory effect on bacterial growth compared with the empty plasmid control (Supplementary Fig. S1).

**Fig. 2:**
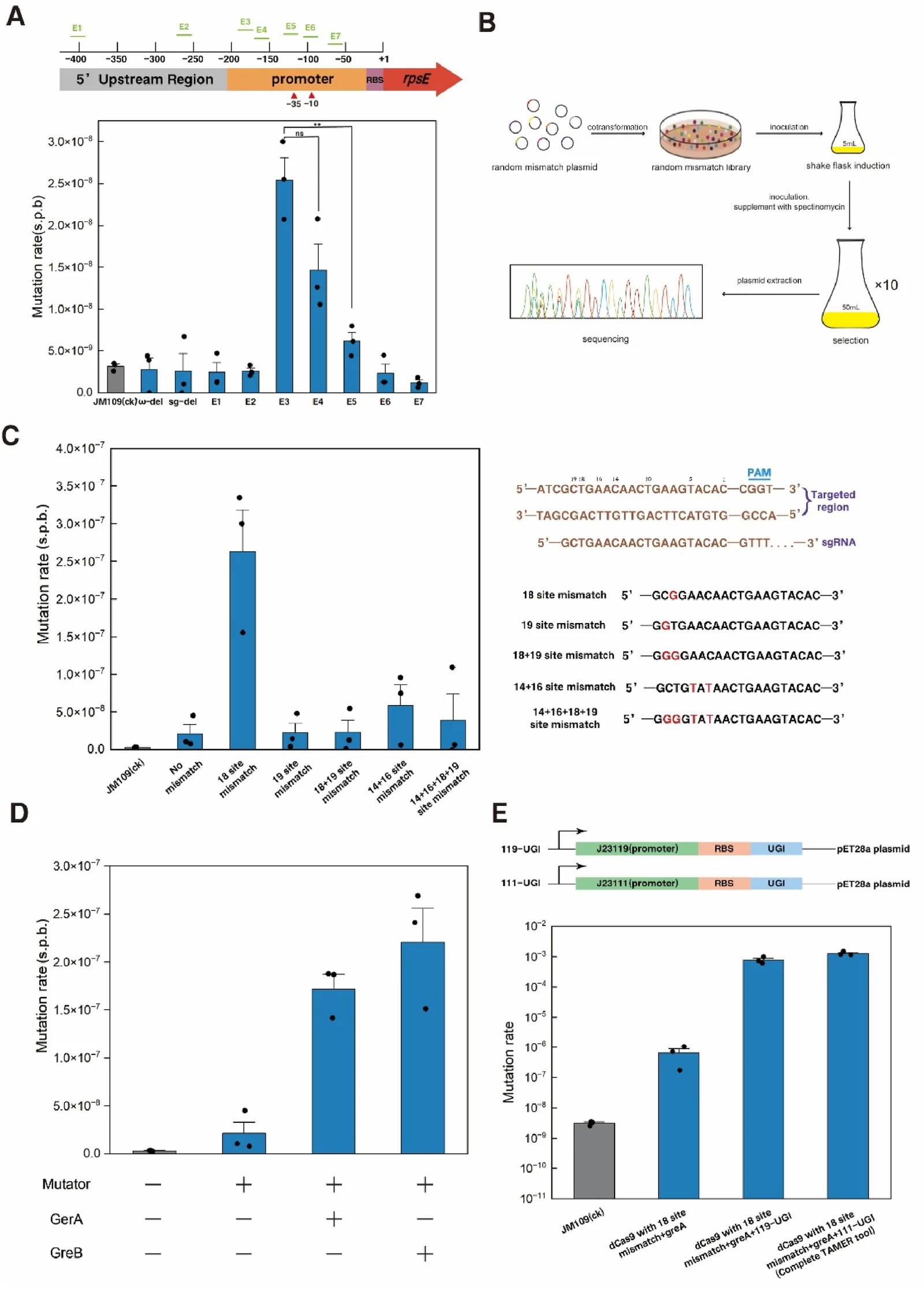
**Optimization of mutagenesis performance in TAMER.** (A) Effect of sgRNA targeting positions on mutagenesis efficiency. (B) Workflow for constructing and screening an sgRNA mismatch library. (C) Influence of sgRNA mismatches at different positions on mutagenesis efficiency. (D) Effect of transcription elongation factors on mutagenesis efficiency. (E) Impact of UGI expression driven by different promoters on mutagenesis efficiency. Data are presented as mean (bar), standard error (error bar), and individual data points (black dots). n = 3 independent biological replicates. NS, not significant (P > 0.05). *P < 0.05, **P < 0.01, ***P < 0.001 and ****P < 0.0001.

Based on this, the present study further optimized the tool from the aspects of transcription initiation and elongation, aiming to enhance its mutation efficiency. To facilitate the effective assembly of RNAP at the target site, multiple distinct sgRNA targeting sites were designed within the 400 bp regions upstream of the *rpsE* gene. Experimental results indicated that when the targeting site was located 160–194 bp upstream of the transcription start site (approximately 39–73 bp upstream of the promoter −35 region), the mutation efficiency reached its maximum level (Fig. 2A). To reduce steric hindrance during transcription initiation, five catalytically inactive Cas protein variants with localization functions, as reported in the literature [[12], [13], [14], [15], [16]], were evaluated. The results showed that when Un1Cas12f1 was employed, the mutation frequency reached 1.1 × 10^−7^ s.p.b., which was approximately 36-fold higher than the natural mutation frequency, however, there was no significant difference in mutation frequency compared with that of dCas9 (Supplementary Fig. 2). Previous studies have indicated that introducing mismatches at positions 14, 16, 18, and 19 of the sgRNA protospacer can significantly reduce the affinity between the Cas protein and DNA without affecting targeting specificity [17]. Therefore, an sgRNA mismatch library covering the aforementioned positions was constructed, and mismatch combinations with advantageous mutant phenotypes were isolated through spectinomycin screening. The results revealed that when a mismatch was introduced at position 18 of the protospacer corresponding to dCas9, the tool achieved the highest mutation efficiency of 2.5 × 10^−7^ s.p.b., approximately 81-fold higher than the natural mutation frequency (Fig. 2B and C). Furthermore, the same sgRNA mismatch design was applied to the *rpsL* gene, and the results showed that a mismatch at position 18 also significantly improved the mutation efficiency, reaching 6.5-fold higher than that without mismatch (Supplementary Fig. 3). In contrast, engineered variants based on literature-reported mismatch sites [18] aimed at reducing the affinity between Un1Cas12f1 and its protospacer failed to enhance mutation frequency, instead resulting in a noticeable decline (Supplementary Fig. 4).

To ensure transcriptional continuity and mitigate transcriptional arrest induced by the fusion proteins, we co-expressed the fusion construct with the transcription elongation factors GreA or GreB, which are known to suppress RNAP backtracking and facilitate transcriptional recovery [[19], [20], [21]]. Experimental evaluations demonstrated that the co-expression of either GreA or GreB substantially enhanced mutation efficiency, reaching 1.3 × 10^−7^ s.p.b. and 2.3 × 10^−7^ s.p.b., respectively—marking a 45-fold and 74-fold increase over the baseline natural mutation rate (Fig. 2D). Building upon these optimization parameters, we combined the targeting strategy at 174 bp upstream of the *rpsE* gene with a position-18 mismatched sgRNA for dCas9, alongside the co-expression of GreB. Under these combined conditions, the mutation rate was further elevated to 4.5 × 10^−7^ s.p.b. (Fig. 2E), representing an approximately 156-fold increase over the natural mutation rate. This outcome validated the efficacy of synergistically optimizing both transcription initiation and elongation. Furthermore, to improve the deamination rate, we sought to enhance the apparent kinetic efficiency of cytidine deamination by co-expressing an uracil glycosylase inhibitor (UGI) [8,22]. To evaluate the impact of varied expression levels on mutation efficiency, UGI expression was driven by either the strong promoter J23119 or the medium-strength promoter J23111. The results indicated that UGI co-expression successfully boosted mutation efficiency, with the J23111 promoter yielding a more pronounced effect. Specifically, the mutation rate reached 1.28 × 10^−3^ s.p.b., which corresponds to an approximately 4.2 × 10^5^-fold increase over the natural mutation rate. Together, systematic engineering of endogenous RNAP transcription (initiation and elongation) and deamination kinetics significantly improved the mutagenesis efficiency of TAMER.

**Fig. 3:**
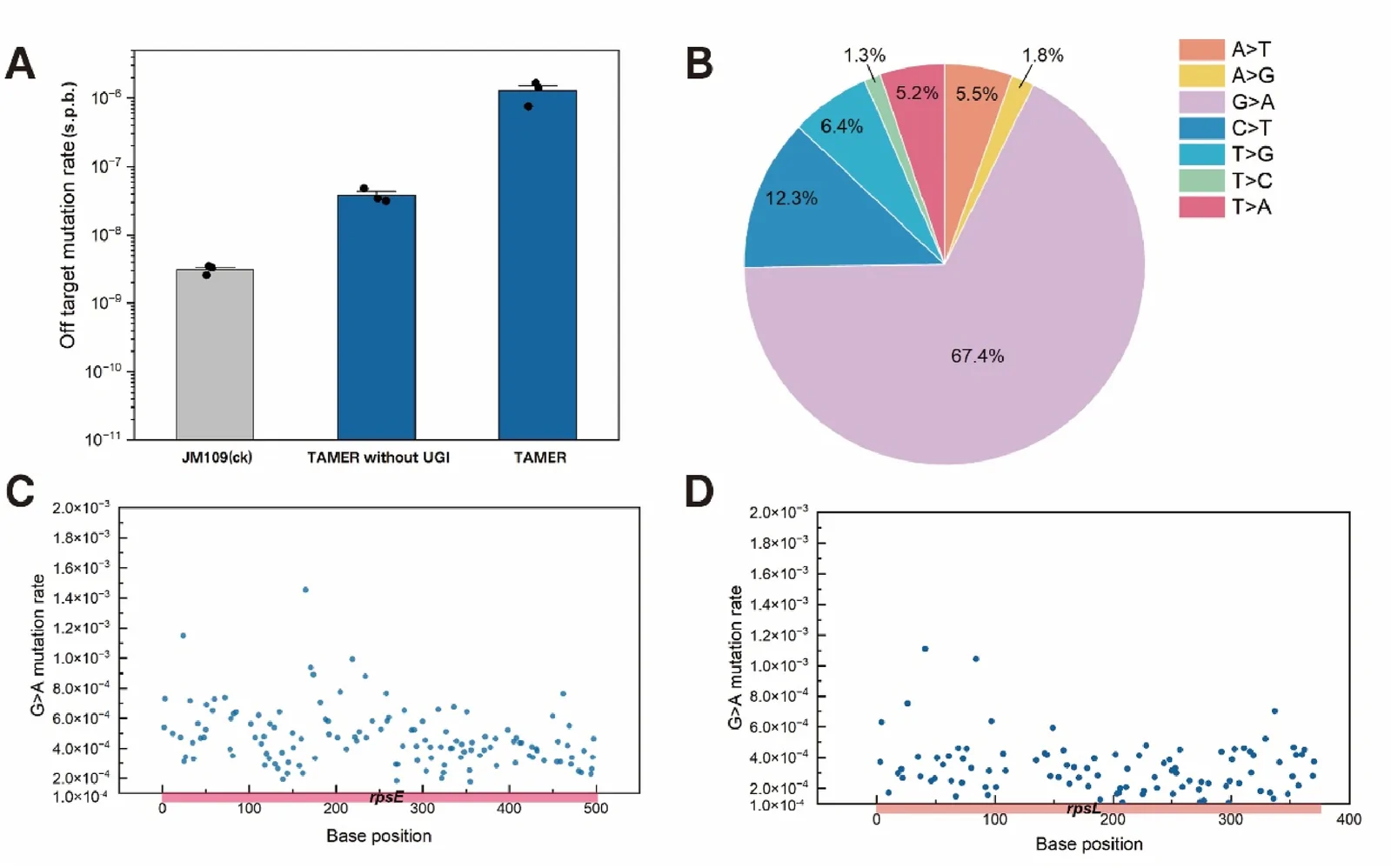
**Characterization of the mutagenesis performance of TAMER.** (A) Genomic off-target mutation frequency. (B) Mutation types and their proportions introduced by TAMER. (C, D) Frequency and positional distribution of G-to-A mutations in the targeted *rpsE* (C) and *rpsL* (D) genes as revealed by NGS sequencing.

Subsequently, a systematic characterization of the mutation performance of TAMER was conducted. Using the classical rifampicin resistance assay (Rif^R^), the mutation frequency in the genomic *rpoB* gene was measured to evaluate the sgRNA-independent off-target effects of the tool. After 24 h, the Rif^R^ frequency was determined. The results indicated that while UGI co-expression enhanced the non-targeted mutation background alongside the increase in targeted mutation frequency, the ratio of targeted to non-targeted mutation frequencies remained above 1000-fold (Fig. 3A). For sgRNA-dependent off-target sites, predictions were performed using Cas-OFFinder, and the predicted sites are listed in Table S4. Deep sequencing of these predicted sites revealed no significant C > T and G > A mutations. These results demonstrate that the tool can effectively confine mutation events to specific genomic regions. Simultaneously, Sanger sequencing analysis of the *rpsE* gene treated with TAMER revealed that the tool could introduce a diverse range of point mutations, exhibiting a broad mutation spectrum. The predominant mutation types were G > A and C > T, consistent with the catalytic properties of the employed CDA deaminase (Fig. 3B).

To further quantify the mutational efficiency, high-throughput sequencing was performed after 24 h of targeted mutagenesis on the *rpsE* and *rpsL* genes. To minimize technical background potentially introduced during sequencing, analysis was restricted to reliable variants with mutation frequencies exceeding 1 × 10^−4^ s.p.b. The results showed that TAMER uniformly introduced mutations across the full length of both genes, with mutation frequencies reaching the order of 10^−3^ s.p.b (Fig. 3C and D). In summary, TAMER demonstrated the following integrated performance advantages: high targeted mutation efficiency, a broad mutation spectrum, a wide and effective mutation window, and uniform distribution of mutation sites within the targeted region.

**Fig. 4:**
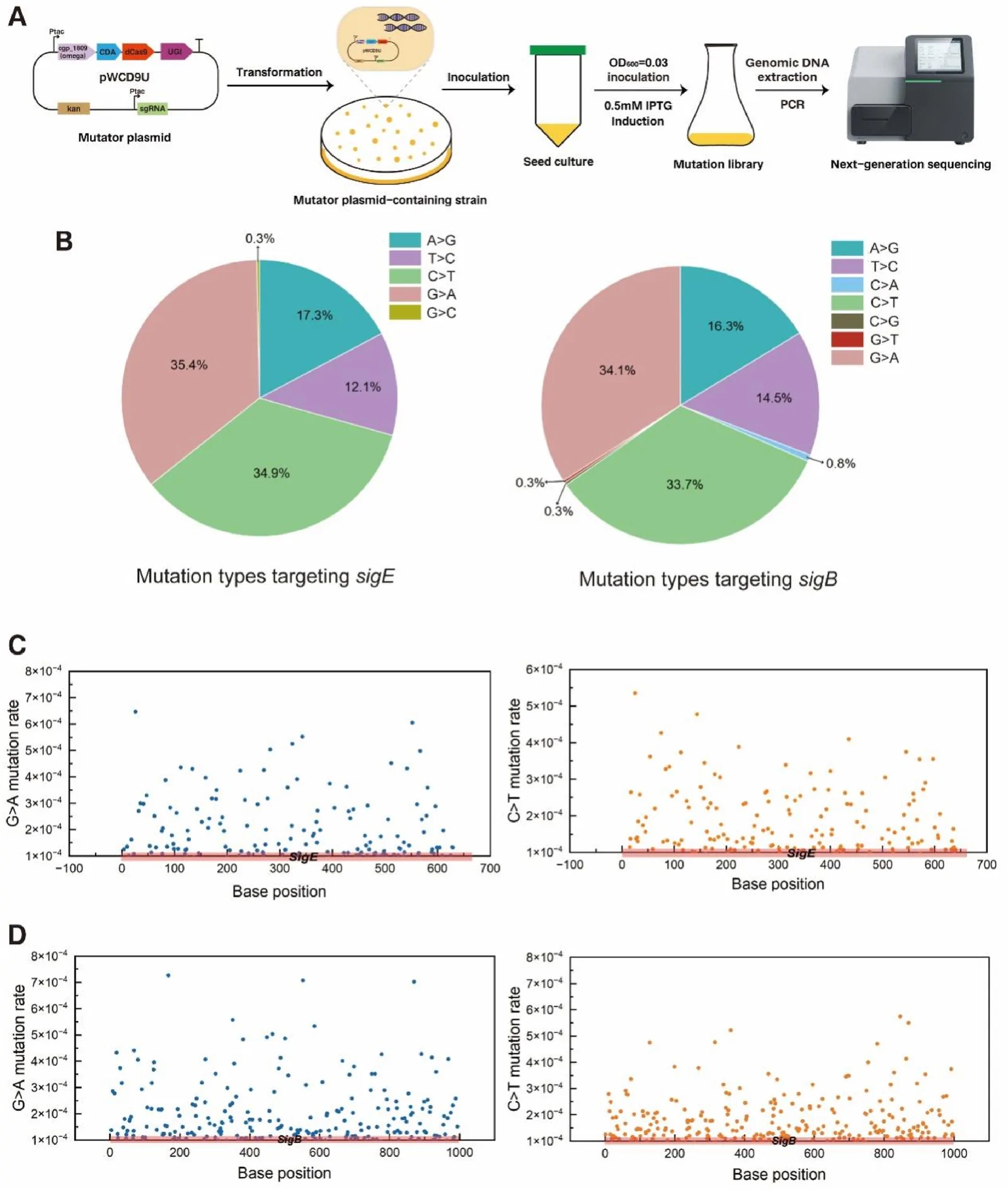
**Validation of the cross-host portability of TAMER.** (A) Reconstruction and validation of TAMER in *C*. *glutamicum*. (B) Distribution of mutation types detected upon targeting the sigE (left) and sigB (right) genes in *C. glutamicum*. (C, D) Frequency and positional distribution of G→A and C→T mutations along the targeted *sigE* (C) and *sigB* (D) genes, as determined by next-generation sequencing (NGS).

To evaluate the cross-host applicability of TAMER, we further characterized its performance in the industrially important chassis *C. glutamicum*. Unlike the strategy employed in *E*. *coli*, a single-plasmid system for co-expression of the mutagenesis apparatus and UGI was used here. Two genes encoding alternative sigma factors in *C. glutamicum*, *sigB* and *sigE*, were selected as targets. Following 24 h of induction with IPTG, deep sequencing was performed (Fig. 4A). Sites with a mutation frequency below 1 × 10^−4^ s.p.b. were excluded from analysis. The sequencing results demonstrated that TAMER predominantly introduced G→A and C→T transitions in *C. glutamicum*, and the mutations were uniformly distributed across the full length of both target genes, with the highest mutation frequency reaching 6.5 × 10^−4^ s.p.b. (Fig. 4B, C, and D). Collectively, TAMER exhibits the following integrated performance advantages: high targeted mutagenesis efficiency, a broad mutagenic window, broad distribution of mutations throughout the target gene, and cross-host portability.

## 3 Discussion

In this study, we developed a novel in vivo targeted mutagenesis platform, termed TAMER, by integrating dCas9, a cytidine deaminase (CDA), and the endogenous RNAP ω subunit. To improve mutagenesis efficiency, we systematically optimized the platform across both the transcription initiation and elongation phases. Initial screening identified that a flexible GSAASR linker between ω and CDA enhanced mutagenesis 6-fold over spontaneous levels. Targeting sgRNA 160-194 bp upstream of the target gene and introducing a mismatch at protospacer position 18 (to attenuate dCas9-DNA binding and reduce sliding resistance) further boosted efficiency to 81-fold above background. Co-expression of the elongation factor GreA (to mitigate RNAP backtracking) and UGI (to accelerate deamination kinetics) synergistically improved performance. The final optimized TAMER (ω-CDA-dCas9, position-18 mismatch, GreA/UGI) achieved a peak mutation frequency of 1.28 × 10^−3^ s.p.b., a 4.2 × 10^5^-fold increase over the natural baseline. In conclusion, TAMER overcomes key limitations of conventional in vivo targeted mutagenesis tools, including narrow windows, uneven distribution, low efficiency, and poor host portability.

Despite these capabilities, TAMER, akin to contemporary targeted diversification technologies, is currently constrained by notable off-target effects. These off-target events can be classified into two categories based on their mechanisms. sgRNA-dependent off-target effects result from sequence homology between the target and other genomic regions, which redirects the mutagenesis complex to non-target loci. sgRNA-independent off-target effects, on the other hand, may involve: (i) association of CDA-fused ω subunits with native transcription complexes, enabling access to transient ssDNA; (ii) action of free or weakly localized CDA on ssDNA generated during replication, transcription, repair, or R-loop formation; and (iii) global UGI-mediated suppression of uracil repair, which stabilizes uracils from both spontaneous deamination and non-specific CDA activity. To mitigate sgRNA-independent off-target effects, future iterations could incorporate stringent spatiotemporal control via inducible promoters, introduce strongly interacting colocalization systems such as SpyTag/SpyCatcher, or spatially couple UGI with the targeting complex to avoid global inhibition of uracil repair. For sgRNA-dependent off-target effects, optimization of sgRNA sequences through genome-wide off-target prediction is recommended, with particular attention paid to avoiding potential off-target sites near active promoter regions. By hijacking host RNA polymerase for processive in situ mutagenesis, TAMER exhibits robust host portability and can, in the future, be designed in other important industrial chassis, even those with difficult genetic manipulation, following standard protocols. Unlike other long-range in situ mutagenesis systems, such as MutaT7 and OTM, TAMER eliminates the tedious requirement of pre-integrating heterologous orthogonal promoter sequences. Instead, it only requires the straightforward design of sgRNAs targeting appropriate positions within endogenous promoter sequences, highlighting the convenience of TAMER. It is precisely this simplicity that makes TAMER more suitable for multiplexed in situ targeted mutagenesis of genes for co-evolution. Error-prone DNA polymerase-driven in situ mutagenesis systems, such as EvolvR, OMEGA-R, and CTRLE, exhibit high mutation rates near the nick site, with mutation rates gradually decreasing as the distance from the nick increases. Next-generation sequencing results show that TAMER introduces uniform mutations at the target positions with a stable mutation rate, indicating the robustness of its mutagenesis. Taken together, TAMER establishes a highly efficient, versatile, and portable paradigm for continuous directed evolution, offering new approaches for next-generation protein engineering, metabolic pathway optimization, and other synthetic biology applications.

## 4 Methods and materials

**Plasmid Construction.** All plasmids were constructed using Gibson assembly and Golden Gate strategies. The editor components were expressed from a pKD vector, which carries an ampicillin resistance gene and enables co-expression of sgRNA and *greA* on the same plasmid. UGI was expressed from a kanamycin-resistant pET-28a vector. Polymerase chain reaction (PCR) was performed using the KOD One™ PCR Master Mix-Blue (Takara), and template removal was carried out with *Dpn*I (Takara). PCR products were purified via column-based cleanup using the DNA Clean-Up Kit (CWBIO). Enzymes employed in ligation reactions were supplied by TransGen Biotech.

**Strain Mutagenesis and Cultivation.** In this study, targeted mutagenesis of the gene of interest was performed by first culturing chemically or electrotransformed *E. coli* JM109 strains on LB agar plates for 24 h. Single colonies were then inoculated into 5 mL of liquid medium and incubated at 30 °C with shaking at 230 rpm for an additional 24 h. The method for quantifying mutation frequency is described below.

**Mutation Frequency Characterization.** In this study, mutation frequency is defined as the ratio of antibiotic-resistant colonies to the total viable bacterial count, calculated as follows:

Mutation Frequency = (Number of Resistant Colonies)/(Total Viable Count)

The total viable count was determined using the dilution plating method. Specifically, bacterial cultures were diluted 10^−6^ -fold, and 100 μL of the diluted suspension was spread onto LB agar plates using a spiral plater. Each sample was processed in three technical replicates. After 24 h of incubation, the colony counts from the three plates were averaged and multiplied by the dilution factor (10^7^) to obtain the total viable count per milliliter. For enumeration of resistant colonies, 1 mL or 0.5 mL of bacterial culture was centrifuged at 6000 rpm for 5 min to pellet the cells. The pellet was resuspended in 300 μL of sterile water, and 100 μL of the resuspension was plated onto agar plates containing 50 mg/mL spectinomycin (or, for off-target mutation frequency assessment, 200 mg/mL rifampicin). Plates were incubated at 30 °C for 48 h before colonies were counted. The sum of colonies from three replicate plates represented the number of resistant colonies per milliliter.

In *E*. *coli*, when the mutation frequency exceeds 10^−4^, the above method for counting resistant colonies may introduce errors due to potential false positives. Therefore, for samples with mutation frequencies above 10^−4^, 100 μL of undiluted culture was directly plated onto plates containing spectinomycin (50 mg/L), with each sample plated in three technical replicates. The average colony count from the three plates was taken as the number of resistant cells. Colony counting was performed after 48 h of plate incubation.

### 4.1 High-throughput sequencing analysis

For sequencing analysis, bacteria were first subjected to targeted mutagenesis in 5 mL of LB medium for 24 h. Genomic DNA was subsequently extracted using a commercial DNA purification kit (TIANGEN DP302) according to the manufacturer's instructions. The isolated DNA was used as template for PCR amplification of the target region(s). Amplified products were purified via column-based cleanup. The purified PCR products were then submitted to GENEWIZ (Beijing, China) for library construction, high-throughput sequencing, and subsequent bioinformatic analysis. The key procedures performed by GENEWIZ are detailed below.

**Library construction and sequencing.** For each sample, purified PCR product (>50 ng) was physically sheared to 200–300 bp fragments. The fragmented DNA was end-repaired, 5′-phosphorylated, and dA-tailed in one reaction, followed by adaptor ligation and purification with DNA Clean Beads. Enrichment PCR was performed using P5 and P7 indexing primers. The final library was purified, qualified, and sequenced on an Illumina NovaSeq 6000 platform with paired-end 150 bp reads. Because the PCR amplicon was sheared prior to sequencing, the short reads originated from randomly distributed positions across the full length of the target gene, enabling accurate assessment of mutation distribution across the entire region.

**Data analysis.** Quality control of sequencing data was performed as follows. Raw reads were first subjected to preliminary statistical analysis, then trimmed using Cutadapt [23] (version 1.9.1) to remove primers and adapters, as well as low-quality bases (quality score < 20) at both ends and reads with an N-base ratio exceeding 10%. The resulting clean reads were then merged into complete sequences using Pandaseq [24] (version 2.7) based on the overlapping regions between Read1 and Read2, and the merged sequences were stored in FASTA format. The length distribution of merged sequences was subsequently calculated for each sample. Reads were then split according to their barcodes, and the split results were tallied. For target-sequence abundance statistics, if a specific target region was defined, sequences were captured using 10 nt of flanking sequence on each side of the target; otherwise, the full-length sequences were used as the reference. Finally, the merged sequences were aligned to the reference sequence using BWA software, and the aligned portion was extracted to count mutation abundance information, allowing the base distribution and mutation frequency to be determined.

## Supporting information

Fig. S1–S4 and Table S1–S4 provide data on bacterial growth under TAMER expression, Cas protein substitution effects, sgRNA mismatch effects (including with UN1Cas12f1), effects of transcription elongation factors, and the lists of plasmids, primers, strains, and predicted potential off-target sites used in this study.

## CRediT authorship contribution statement

**Yuou Sheng:** Writing – original draft, Visualization, Validation, Investigation, Formal analysis, Data curation, Conceptualization. **Huizhen Ni:** Validation, Investigation, Data curation. **Shihao Yang:** Visualization, Formal analysis, Data curation, Conceptualization. **Wenliang Hao:** Writing – review & editing, Supervision, Data curation, Conceptualization. **Chong Zhang:** Writing – review & editing, Supervision, Resources, Project administration, Funding acquisition.

## Data availability statement

The raw sequencing data have been submitted to the NCBI SRA database and are accessible under accession number [PRJNA1517198] upon reasonable request.

## Declaration of competing interest

The authors declare that they have no known competing financial interests or personal relationships that could have appeared to influence the work reported in this paper.
