# Supplementary material for: Hijacking host RNA polymerase for processive in situ mutagenesis
Source: Synth Syst Biotechnol. 2026 Sep 18;18:142–9. doi: 10.1016/j.synbio.2026.09.002 (PMC13602049; doi:10.1016/j.synbio.2026.09.002)
Supplement: Supplementary file 1 [file mmc1.docx]

**Hijacking Host RNA Polymerase for Processive In Situ Mutagenesis**

Yuou Sheng^a^, Huizhen Ni^a^, Shihao Yang^b^, Wenliang Hao^a,c^*, and Chong Zhang^a,d,e,f^*

^a^MOE Key Laboratory for Industrial Biocatalysis, Institute of Biochemical Engineering, Department of Chemical Engineering, Tsinghua University, Beijing 100084, China

^b^School of Life Sciences, Tsinghua University, Beijing 100084, China

^c^College of Food Science and Technology, Nanjing Agricultural University, Nanjing, 210095, PR China

^d^Beijing Key Laboratory of Recombinant Proteins and Biomanufacturing, Tsinghua University, Beijing 100084, China

^e^State Key Laboratory of Green Biomanufacturing, Tsinghua University, Beijing 100084, China

^f^Center for Synthetic and Systems Biology, Tsinghua University, Beijing 100084, China

*Corresponding author: Wenliang Hao, Email: [wenlianghao@njau.edu.cn](mailto:wenlianghao@njau.edu.cn). Chong Zhang, Email: [chongzhang@tsinghua.edu.cn](mailto:chongzhang@tsinghua.edu.cn).

**Contents**

**Supplementary Figure 1**: The impact of the TAMER system on growth.	3

**Supplementary Figure 2:** The impact of different Cas proteins on the mutagenesis efficiency of TAMER.	4

**Supplementary Figure 3:** Impact of sgRNA protospacer mismatches on mutagenesis efficiency in the UN1Cas12f1‑based TAMER system.	5

**Supplementary Figure 4:**Impact of sgRNA protospacer mismatch position targeting the *rpsL* gene on mutagenesis efficiency.	6

**Supplementary Figure 5:** Effects of sgRNA protospacer mismatches and transcription elongation factors on mutagenesis efficiency in the TAMER system.	7

**Table S1: Plasmid used in this study.**	8

**Table S2: Primers used in this study.**	12

**Table S3: Strains used in this study.**	18

**Table S4: sgRNA-dependent off-target sites predicted by Cas-offinder**	19

**Supplementary Figure 1**: The impact of the TAMER system on growth.

**Supplementary Figure 2:** The impact of different Cas proteins on the mutagenesis efficiency of TAMER.

**Supplementary Figure 3:** Impact of sgRNA protospacer mismatch position targeting the *rpsL* gene on mutagenesis efficiency.

**Supplementary Figure 4:** Impact of sgRNA protospacer mismatches on mutagenesis efficiency in the UN1Cas12f1‑based TAMER system.

**Supplementary Figure 5:** Effects of sgRNA protospacer mismatches and transcription elongation factors on mutagenesis efficiency in the TAMER system.

Table S1 Plasmid used in this study

| Plasmid | Relevant characteristic | Reference |
| --- | --- | --- |
| pKD-$\alpha$-CDA-dCas9 | Expression of RpoA–PmCDA1–dCas9 fusion protein with sgRNA targeting the upstream region of the *rpsE* gene. | This study |
| pKD-$\beta$-CDA-dCas9 | Expression of RpoB–PmCDA1–dCas9 fusion protein with sgRNA targeting the upstream region of the *rpsE* gene. | This study |
| pKD-$\beta’$-CDA-dCas9 | Expression of RpoC–PmCDA1–dCas9 fusion protein with sgRNA targeting the upstream region of the *rpsE* gene. | This study |
| pKD-ω-CDA-dCas9-GSAASR | Expression of RpoZ–PmCDA1–dCas9 fusion protein with a GSAASR linker and sgRNA targeting the *rpsE* gene. | This study |
| pKD-ω-CDA-dCas9-AP2 | Expression of RpoZ–PmCDA1–dCas9 fusion protein with a (AP)_2_ linker and sgRNA targeting the *rpsE* gene. | This study |
| pKD-ω-CDA-dCas9-AEK2 | Expression of RpoZ–PmCDA1–dCas9 fusion protein with a (AEK)_2_ linker and sgRNA targeting the *rpsE* gene. | This study |
| pKD-ω-CDA-dCas9-E1 | Expression of RpoZ–PmCDA1–dCas9 fusion protein with sgRNA targeting a site 394 bp upstream of the *rpsE* gene (sg‑E1). | This study |
| pKD-ω -CDA-dCas9-E2 | Expression of RpoZ–PmCDA1–dCas9 fusion protein with sgRNA targeting a site 256 bp upstream of the *rpsE* gene (sg‑E2). | This study |
| pKD-ω-CDA-dCas9-E3 | Expression of RpoZ–PmCDA1–dCas9 fusion protein with sgRNA targeting a site 174 bp upstream of the *rpsE* gene (sg‑E3). | This study |
| pKD-ω-CDA-dCas9-E4 | Expression of RpoZ–PmCDA1–dCas9 fusion protein with sgRNA targeting a site 160 bp upstream of the *rpsE* gene (sg‑E4). | This study |
| pKD-ω-CDA-dCas9-E5 | Expression of RpoZ–PmCDA1–dCas9 fusion protein with sgRNA targeting a site114 bp upstream of the *rpsE* gene (sg‑E5). | This study |
| pKD-ω-CDA-dCas9-E6 | Expression of RpoZ–PmCDA1–dCas9 fusion protein with sgRNA targeting a site 83 bp upstream of the *rpsE* gene (sg‑E6). | This study |
| pKD-ω-CDA-dCas9-E7 | Expression of RpoZ–PmCDA1–dCas9 fusion protein with sgRNA targeting a site 52 bp upstream of the *rpsE* gene (sg‑E7). | This study |
| pKD-CDA-dCas9-E3 | Expression of the PmCDA1‑dCas9 fusion protein with sgRNA, without RpoZ. | This study |
| pKD-sgRNA-E3 | Expression of sgRNA targeting a site 174 bp upstream of the *rpsE* gene, without the mutagenesis system. | This study |
| pKD-EC | The pKD plasmid vector expressing neither the mutagenesis system nor sgRNA. | This study |
| pKD-ω-CDA-dCas12j-8 | Expression of the RpoZ–PmCDA1–dCas12j‑8 fusion protein with sgRNA targeting a site 170 bp upstream of the *rpsE* gene. | This study |
| pKD-ω-CDA-dCas12m | Expression of the RpoZ–PmCDA1–dCas12m fusion protein with sgRNA targeting a site 183 bp upstream of the *rpsE* gene. | This study |
| pKD-ω-CDA-dCWCas12f | Expression of the RpoZ–PmCDA1‑dCWCas12f fusion protein with sgRNA targeting a site 172 bp upstream of the *rpsE* gene. | This study |
| pKD-ω-CDA-dIscBQM | Expression of the RpoZ–PmCDA1–dIscBQM fusion protein with sgRNA targeting a site 168 bp upstream of the *rpsE* gene. | This study |
| pKD-ω-CDA-dUn1Cas12f1 | Expression of the RpoZ–PmCDA1–dUn1Cas12f1 fusion protein with sgRNA targeting a site 168 bp upstream of the *rpsE* gene (sg‑Un). | This study |
| pKD-ω-CDA-dCas9-18 | Expression of RpoZ–PmCDA1–dCas9 fusion protein with sg‑E3 containing a single‑nucleotide mismatch 18 bp from the PAM sequence. | This study |
| pKD-ω-CDA-dCas9-19 | Expression of RpoZ–PmCDA1–dCas9 fusion protein with sg‑E3 containing a single‑nucleotide mismatch 19 bp from the PAM sequence. | This study |
| pKD-ω-CDA-dCas9-1819 | Expression of RpoZ–PmCDA1–dCas9 fusion protein with sg‑E3 containing dual mismatches at positions 18 bp and 19 bp from the PAM sequence. | This study |
| pKD-ω-CDA-dCas9-1614 | Expression of RpoZ–PmCDA1–dCas9 fusion protein with sg‑E3 containing mismatches at positions 14 bp and 16 bp from the PAM sequence. | This study |
| pKD-ω-CDA-dCas9-4m | Expression of RpoZ–PmCDA1–dCas9 fusion protein with sg‑E3 containing mismatches at positions 14, 16, 18, and 19 bp from the PAM sequence. | This study |
| pKD-ω-CDA-dUn1Cas12f1-2 | Expression of RpoZ–PmCDA1–dUn1Cas12f1 fusion protein with sg‑Un containing a mismatch 2 bp from the PAM sequence. | This study |
| pKD-ω-CDA-dUn1Cas12f1-6 | Expression of RpoZ–PmCDA1–dUn1Cas12f1 fusion protein with sg‑Un containing a mismatch 6 bp from the PAM sequence. | This study |
| pKD-ω-CDA-dUn1Cas12f1-18 | Expression of RpoZ–PmCDA1–dUn1Cas12f1 fusion protein with sg‑Un containing a mismatch 18 bp from the PAM sequence. | This study |
| pKD-ω-CDA-dUn1Cas12f1-19 | Expression of RpoZ–PmCDA1–dUn1Cas12f1 fusion protein with sg‑Un containing a mismatch 19 bp from the PAM sequence. | This study |
| pKD-ω-CDA-dCas9-GreA | Expression of RpoZ–PmCDA1–dCas9 fusion protein, sg‑E3, and the transcription elongation factor GreA. | This study |
| pKD-ω-CDA-dCas9-GreB | Expression of RpoZ–PmCDA1–dCas9 fusion protein, sg‑E3, and the transcription elongation factor GreB. | This study |
| pKD-ω-CDA-dCas9-18-GreA | Expression of RpoZ–PmCDA1–dCas9 fusion protein, sg‑E3 with an 18 bp PAM‑distal mismatch, and the transcription elongation factor GreA. | This study |
| pKD-ω-CDA-dCas9-18-GreB | Expression of RpoZ–PmCDA1–dCas9 fusion protein, sg‑E3 with an 18 bp PAM‑distal mismatch, and the transcription elongation factor GreB. | This study |
| pET28a-J23119-UGI | Expression of UGI driven by the J23119 promoter. | This study |
| pET28a-J23111-UGI | Expression of UGI driven by the J23111 promoter. | This study |
| pKD-ω-CDA-dCas9-RpoB | Expression of RpoZ–PmCDA1–dCas9 fusion protein with sgRNA targeting the *rpoB* gene. | This study |
| PWCD9U-TAMER-C.g | Expression of TAMER tool in *Corynebacterium glutamicum* | This study |

Table S2 Primers used in this study

| prime | Sequence (5’-3’) | purpose |
| --- | --- | --- |
| rpoA-F | ctctaaggaggttataaaaaatgcagggttctgtgacagag | Forward primer for rpoA (α subunit) cloning |
| rpoA-R | GTtctagaagctgcagaaccttactcgtcagcgatgcttgc | Reverse primer for rpoA (with linker overlap) |
| rpoB-F | ctctaaggaggttataaaaaatggtttactcctataccgagaaaaaacgt | Forward primer for rpoB (β subunit) cloning |
| rpoB-R | GTtctagaagctgcagaaccttactcgtcttccagttcgatgttg | Reverse primer for rpoB (with linker overlap) |
| rpoC-F | tctaaggaggttataaaaaAtgaaagatttattaaagtttctgaaagcgca | Forward primer for rpoC (β' subunit) cloning |
| rpoC-R | GTtctagaagctgcagaaccttactcgttatcagaaccgccc | Reverse primer for rpoC (with linker overlap) |
| rpoZ-F | gttataaaaaatgttagatccgtcaattgattctttaatg | Forward primer for rpoZ (ω subunit) cloning |
| rpoZ-R | tctagaagctgcagaaccttcgcggtcttccttttcaaac | Reverse primer for rpoZ (with linker overlap) |
| P-RNAP-F | tttttataacctccttagagctcgaa | Forward primer for different submit |
| P-RNAP-R | ggttctgcagcttctagaACAGATGCCGAATACGTTCG | Reverse primer for different submit |
| Linker-GSAASR-F | CTTTCGTTTGAAAAGGAAGACCGCGAA  ggttctgcagcttctagaACAGATGCCGAATACGTTCGC | Amplification of PmCDA1 with GSAASR linker |
| Linker-AP2-F | CTTTCGTTTGAAAAGGAAGACCGCGAA  GCGCCGGCGCCG ACAGATGCCGAATACGTTCGC | Amplification of PmCDA1 with (AP)2 linker |
| Linker-AEK2-F | CTTTCGTTTGAAAAGGAAGACCGCGAA  GCGGAAAAAG CGGAAAAA ACAGATGCCGAATACGTTCGC | Amplification of PmCDA1 with (AEK)2 linker |
| sg-E1-F | Tcgtgttgtacaataaatgtcgaagtcgtgcgtaccaaag  gttttagagctagaaatagcaagttaaaataagg | Forward primer for sg-E1 (-394 bp) |
| sg-E2-F | Tcgtgttgtacaataaatgtgaagaagtaaggtaacacta  gttttagagctagaaatagcaagttaaaataagg | Forward primer for sg-E2 (-256 bp) |
| sg-E3-F | Tcgtgttgtacaataaatgtccgcgcacgccgcaagctcc  gttttagagctagaaatagcaagttaaaataagg | Forward primer for sg-E3 (-174 bp) |
| sg-E4-F | TcgtgttgtacaataaatgtGTACACCGGTAACAAAGACG  gttttagagctagaaatagcaagttaaaataagg | Forward primer for sg-E4 (-160 bp) |
| sg-E5-F | Tcgtgttgtacaataaatgtcccgcgtcacatttacgcac  gttttagagctagaaatagcaagttaaaataagg | Forward primer for sg-E5 (-114 bp) |
| sg-E6-F | Tcgtgttgtacaataaatgt gctgaacgcgctctggaaaa  gttttagagctagaaatagcaagttaaaataagg | Forward primer for sg-E6 (-84 bp) |
| sg-E7-F | Tcgtgttgtacaataaatgt gatgtatcctttgaccgttc gttttagagctagaaatagcaagttaaaataagg | Forward primer for sg-E7 (-52 bp) |
| sg-Universal-R | ACATTTATTGTACAACACGAGCCCATT | Universal Reverse primer for sgRNA cloning |
| dCas12j8-F | agtctgcaactccagagtctATGATCAAACCGACCGTTTCT | Forward primer for dCas12j-8 coding sequence |
| dCas12m-F | agtctgcaactccagagtctATGACCACTATGACTGTTCACAC | Forward primer for dCas12m coding sequence |
| dCWCas12f-F | agtctgcaactccagagtctATGGGTGAGAAATCCAGCC | Forward primer for dCWCas12f coding sequence |
| dIscBQM-F | agtctgcaactccagagtctATGATGGCGGTTGTTTACGT | Forward primer for dIscBQM coding sequence |
| dUn1Cas12f1-F | agtctgcaactccagagtctATGGCGAAGAACACCATCAC | Forward primer for dUn1Cas12f1 coding sequence |
| dCas12j8-R | ttgttagcagccggatcttaAACCGCTTCACGCAGAA | Reverse primer for dCas12j-8 coding sequence |
| dCas12m-R | ttgttagcagccggatcttaTGGATTAGACGGTGCGG | Reverse primer for dCas12m coding sequence |
| dCWCas12f-R | ttgttagcagccggatcttaTGGTTCTTCTTTGGTAGATTTCAGT | Reverse primer for dCWCas12f coding sequence |
| dIscBQM-R | ttgttagcagccggatcttaAACGTAGATCTGCAGACCAC | Reverse primer for dIscBQM coding sequence |
| dUn1Cas12f1-R | ttgttagcagccggatcttaTGGTTCTTCTTTGGTAGACTTC | Reverse primer for dUn1Cas12f1 coding sequence |
| P-Cas-F | taaGATCCGGCTGCTAACAAAG | Forward primers for Cas protein granule skeleton |
| P-Cas-R | agactctggagttgcagactc | Reverse primers for Cas protein granule skeleton |
| Mut-dCas9-18-F | TTGTACAATAAATGTgcGgaacaactgaagtacacgttttagag | Intro of 18 bp mismatch in sg-E3 |
| Mut-dCas9-19-F | TTGTACAATAAATGTggtgaacaactgaagtacacgttttagag | Intro of 19 bp mismatch in sg-E3 |
| Mut-dCas9-1819-F | TTGTACAATAAATGTggggaacaactgaagtacacgttttagagc | Intro of 18,19 bp dual mismatches |
| Mut-dCas9-1614-F | TTGTACAATAAATGTgctgTaTaactgaagtacacgttttagagctaga | Intro of 14,16 bp dual mismatches |
| Mut-dCas9-4m-F | TTGTACAATAAATGTgGGgTaTaactgaagtacacgttttagagctaga | Intro of 14, 16, 18, 19 bp 4-mismatches |
| Mut-dUn-2-F | ATTTGAATGAAGGAATGCAAC  tgaccggtgtacttcagttgCCCGTGACAGGTCATTCAGA | Intro of 2 bp mismatch from PAM (for dUn1Cas12f1) |
| Mut-dUn-6-F | ATTTGAATGAAGGAATGCAAC  ttacctgtgtacttcagttgCCCGTGACAGGTCATTCAGA | Intro of 6 bp mismatch from PAM (for dUn1Cas12f1) |
| Mut-dUn-18-F | ATTTGAATGAAGGAATGCAAC  ttaccggtgtacttcaggtgCCCGTGACAGGTCATTCAGA | Intro of 18 bp mismatch from PAM (for dUn1Cas12f1) |
| Mut-dUn-19-F | ATTTGAATGAAGGAATGCAAC  ttaccggtgtacttcagtggCCCGTGACAGGTCATTCAGA | Intro of 19 bp mismatch from PAM (for dUn1Cas12f1) |
| GreA-F | acaggcgccttttatggattcgccctataaagtaaacgatgacccttc | Forward primer to clone greA from E. coli |
| GreA-R | atgggtagtttccttgcatg ttacaggtattccaccttaattacttcaaattctacttcg | Reverse primer to clone greA from E. coli |
| GreB-F | acaggcgccttttatggattATTTGTAAGCG  TGTATTCTTAAAAAAGCTC | Forward primer to clone greB from E. coli |
| GreB-R | atgggtagtttccttgcatgTTAC  GGTTTCACGTACTCGATAGC | Reverse primer to clone greB from E. coli |
| P-gre-F | 、ttttatggattcatgcaaggaaactaccc | Forward primers for greA/B granule skeleton |
| P-gre-R | ggcgcctgtagtgccatttac | Reverse primers for greA/B granule skeleton |
| J23119-UGI-F | CGACGATAGTCATGCCCCGttgacggctagctcagtcctag | Forward primer with J23119 promoter sequence |
| J23111-UGI-F | CGACGATAGTCATGCCCCGttgacAgctagctcagtcctag | Forward primer with J23111 promoter sequence |
| UGI-R | CAGCTTCCTTTCGGGCTTTGT  TAAAGCATTTTGATCTTATTCTCGCCG | Reverse primer for UGI cloning into pET28a |
| P-UGI-F | CAAAGCCCGAAAGGAAGCTG | Forward primers for UGI granule skeleton |
| P-UGI-R | CGGGGCATGACTATCGTCG | Reverse primers for UGI granule skeleton |
| TAMER-F | AGGACAACTAgtgaccaacgtgagcaacgag | Forward primers for ERDISM in *Corynebacterium glutamicum* |
| TAMER-R | tactgGTACCtcacagcattttgattttgttctcccc | Reverse primers for ERDISM in *Corynebacterium glutamicum* |
| P-ER-F | accgagtcggtgcttttttgGATCCTCTAGAGTCGACCTGCAGG | Forward primers for ERDISM granule skeleton in *Corynebacterium glutamicum* |
| P-ER-R | tcgttgctcacgttggtcacTAGTTGTCCTCCTTTgaattCCATGGTC | Reverse primers for ERDISM granule skeleton in *Corynebacterium glutamicum* |
| Sg-F | aatgctgtgaGGTACcagtagggaactgcc | Forward primers for sgRNA in *Corynebacterium* |
| Sg-R | caaaaaagcaccgactcggtgc | Reverse primers for sgRNA in *Corynebacterium* |

Table S3 Strains used in this study

| Strain Name | Description | Source |
| --- | --- | --- |
| *Escherichia coli* JM109 | Host strain for cloning and targeted sliding mutagenesis characterization | Laboratory stock |
| *Corynebacterium glutamicum* MB001 | Host strain for cross-host portability validation of TAMER | Laboratory stock |

Table S4 sgRNA-dependent off-target sites predicted by Cas-offinder

| Bulge type | crRNA | DNA | Chromosome | Position | Direction | Mismatches | Bulge Size |
| --- | --- | --- | --- | --- | --- | --- | --- |
| DNA | GCG-GAACAACTGAAGTACACNGG | cgcTGAACAACTGAAGTACACCGG | E.coli_K-12 | 1196228 | + | 3 | 1 |
| DNA | GCGGAACA  ACTGAAGTACACNGG | GCGGAAgAACTGgAGTcCAgGGG | E.coli_K-12 | 2169074 | + | 4 | 0 |
